# Photo Quiz: A camper with fever, headache, and fatigue

**DOI:** 10.1128/jcm.01370-25

**Published:** 2026-01-14

**Authors:** Benjamin von Bredow, Matthew D. Sims, Bobby L. Boyanton

**Affiliations:** 1 Department of Pathology, Oakland University William Beaumont School of Medicine 159878, Rochester, Michigan, USA; 2 Department of Pathology & Laboratory Medicine, Corewell Health William Beaumont University Hospital 21818, Royal Oak, Michigan, USA; 3 Departments of Internal Medicine and Foundational Medical Studies, Oakland University William Beaumont School of Medicine 159878, Rochester, Michigan, USA; 4 Section of Infectious Diseases and International Medicine, Department of Medicine, Corewell Health William Beaumont University Hospital 21818, Royal Oak, Michigan, USA; 5 Department of Pathology & Laboratory Medicine, University of Arkansas for Medical Sciences 12215, Little Rock, Arkansas, USA; 6 Department of Pathology & Laboratory Medicine, Arkansas Children’s Hospital, Little Rock, Arkansas, USA; Mayo Clinic Minnesota, Rochester, Minnesota, USA

## PHOTO QUIZ 

A 38-year-old female developed fatigue, headache, nausea, vomiting, diarrhea, and abdominal pain 1 week prior to admission. She spent the last 3 months camping in upstate New York. She occasionally used alcohol and marijuana but denied contact with domestic or wild animals. Mosquito bites were frequent, but she does not recall being bitten by other insects or arthropods. Her symptoms escalated with yellowing of the skin and episodes of hyperthermia (Tmax 38.9°C) and hypothermia (Tmin 33.3°C). She arrived at the emergency department with fever (39.7°C) and mild tachycardia (110 bpm). Physical examination was significant for lethargy and generalized jaundice, including scleral icterus. Past medical history was significant for splenectomy 4 years prior due to lymphoma; she was currently in remission and not receiving chemotherapy.

**Fig 1:**
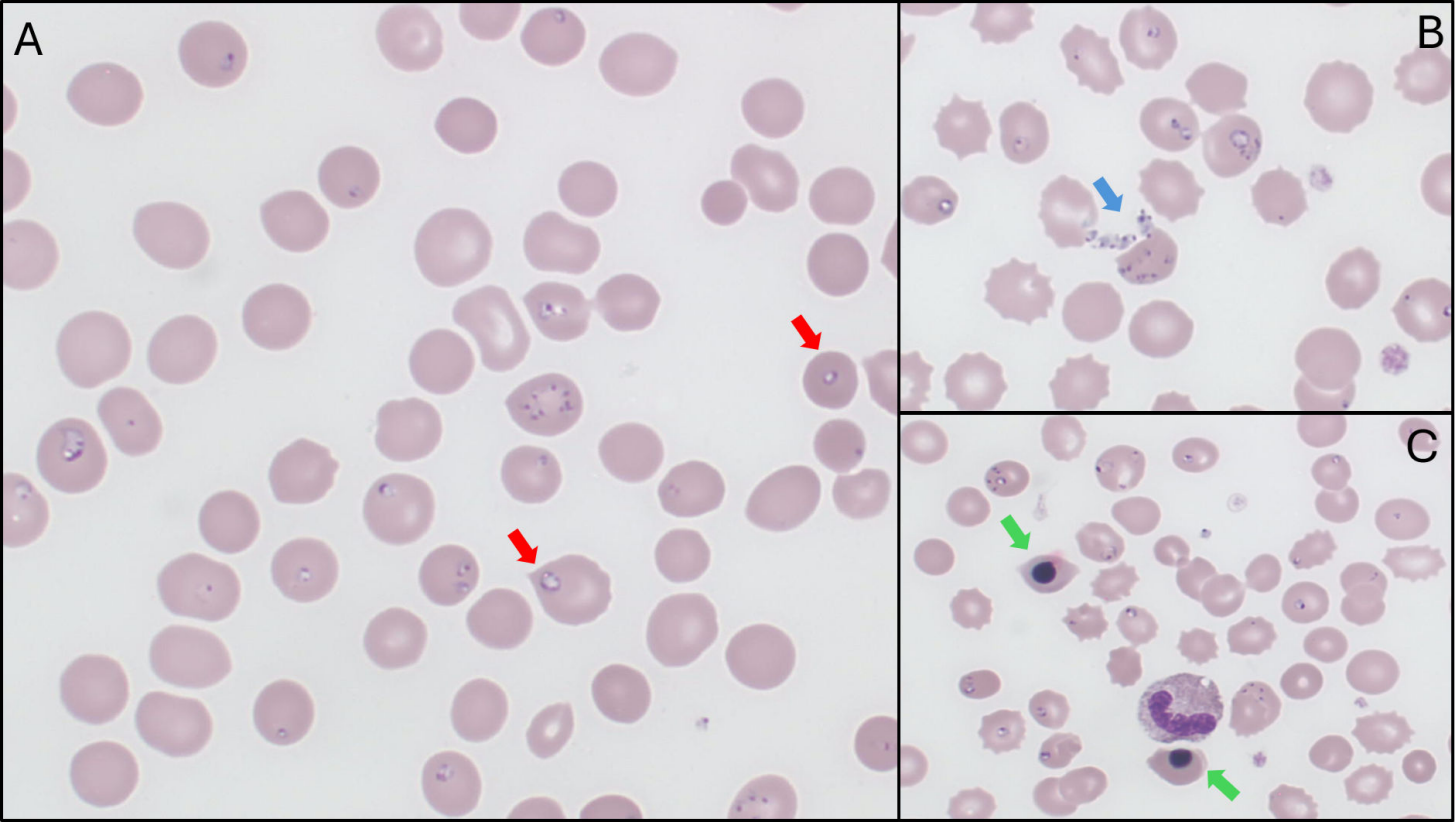
Peripheral blood smear (Wright-Giemsa, 500×). (**A**) Intraerythrocytic ring forms with variation in the number per infected cell (quantity 1–10), size, and shape, with some having intracytoplasmic vacuoles (red arrows). (**B**) Extracellular forms (blue arrow). (**C**) Presence of nucleated red blood cells (green arrows) reflects physiologic compensation for the ongoing hemolytic anemia.

A complete blood count showed an elevated white blood cell count of 13.4 10^9^/L (normal 3.3–10.7) with neutrophilia of 93% (normal 49–67), hemoglobin of 96 g/L (normal 121–150), and platelets of 64 10^9^/L (normal 150–400). Serum studies were significant for BUN of 31.42 mmol/L (normal 0.11–0.43), creatinine of 340.34 µmol/L (normal 53.04–123.76), alanine aminotransferase of 61 U/L (normal 8–37), aspartate aminotransferase of 263 U/L (normal 10–37), lactate dehydrogenase of 2,598 U/L (normal 100–238), total bilirubin of 379.62 µmol/L (normal 5.13–20.52), direct bilirubin of 290.7 µmol/L (normal 0–5.13), and haptoglobin of <707.20 µmol/L (normal 3,536–21,216). Urinalysis was significant for the presence of bilirubin. No pathogens were recovered from blood, urine, and respiratory cultures. A peripheral blood smear was significant for the microorganisms depicted in Fig. 1.

## ANSWER TO PHOTO QUIZ

The patient was diagnosed with babesiosis. She was treated with clindamycin, quinine, and doxycycline, the latter for potential coinfection with other tick-borne diseases. Following three erythrocyte exchange transfusions, parasitemia values dropped from 43 to 7.2, 6.2, and 1.1% over 48 h. Serologic testing (IgM, IgG) for anaplasmosis, ehrlichiosis, and Lyme disease was negative. She responded to antimicrobial therapy and fully recovered.

Babesiosis is a parasitic infection due to protozoans belonging to the genus *Babesia* (1). Eight species are associated with human disease worldwide (2, 3). The estimated global prevalence of human infection is 2.23%, with the highest and lowest rates observed in Europe and North America, respectively (2). Up to 50% of infections are asymptomatic, and they are commonly confused with malaria due to non-specific features, such as fever, fatigue, and hemolytic anemia (2). *Babesia divergens* and *Babesia venatorum* are common causes of babesiosis in Europe and Asia, respectively (2). In North America, *Babesia microti* accounts for most human cases and is transmitted by the definitive host *Ixodes scapularis* (black-legged tick or deer tick); white-footed mice and other small rodents are intermediate hosts. It is endemic in the northeastern and northern midwest regions of the United States (3). Other *Babesia* species are less commonly observed in the United States: *B. duncani* in California and the Pacific Northwest; *B. divergens*-like (MO1) infections in Arkansas, Kentucky, Michigan, Missouri, and Washington; and *B. odocoilei* throughout North America extending south to Texas and north to Saskatchewan (3, 4). Based upon infection acquisition location, *B. microti* was the most likely pathogen, and confirmatory testing was not performed on our patient. However, PCR- or DNA sequencing-based confirmatory testing is encouraged to obtain species-level identification to delineate the actual prevalence of different *Babesia* species causing human infection.

*Babesia* undergoes three reproductive stages: gamogony (sexual) and sprogony (asexual) in the tick gut and salivary glands, respectively; and merogony (asexual) in vertebrate hosts (1). Clinical symptoms primarily include malaise, chills, myalgia, anemia, fatigue, and fever. In severe infection, nausea, vomiting, night sweats, jaundice, and hepatosplenomegaly are observed. Laboratory studies usually demonstrate a normochromic normocytic anemia associated with mild thrombocytopenia and leukocytosis. Serum studies show elevated transaminases, alkaline phosphatase, lactate dehydrogenase, unconjugated bilirubin, BUN, and creatinine (1). Although PCR-based testing is becoming more commonplace, peripheral blood smear microscopy remains the primary diagnostic tool and must be used for parasitemia determination. Observing intraerythrocytic ring forms that vary greatly in number (1 to 10 per cell), size (tiny to large), and shape (round, oval, pyriform), intracytoplasmic vacuoles, extracellular organisms, and the absence of hemozoin pigment are diagnostic features of babesiosis (1). The pathognomonic “Maltese cross” may not be seen due to the ease of diagnosing this particular pathogen based upon the aforementioned diagnostic microscopic features. The primary differential diagnosis is *Plasmodium falciparum* malaria due to elevated parasitemia values (>1–10%) and the morphology of some ring forms. However, marked variability in the number, size, and shape of the ring forms and absence of hemozoin pigment in conjunction with travel history should aid in the correct identification of babesiosis. Treatment includes a combination of clindamycin and quinine or atovaquone and azithromycin. Although controversial, erythrocyte exchange transfusion is still utilized in patients with severe disease, including parasitemia ≥10%, severe hemolysis, and/or organ failure (3, 5).

In summary, babesiosis is a tick-borne zoonosis with worldwide distribution. Infections are generally mild but can be life-threatening in the elderly or immunocompromised, especially asplenic individuals. Blood transfusion and organ transplantation history should also be ascertained, as a small percentage of patients acquire disease via these routes.
